# Neuronal Intranuclear Inclusion Disease with a Corneal Disorder: A Case Report

**DOI:** 10.3390/medicina60111730

**Published:** 2024-10-22

**Authors:** Mohamed Talaat Mohamed, Daisuke Inoue, Shunsuke Yoshimura, Masafumi Uematsu, Yasser Helmy Mohamed, Mao Kusano, Diya Tang, Akio Oishi, Takashi Kitaoka, Gou Takeo, Akihiro Ohira

**Affiliations:** 1 Department of Ophthalmology and Visual Sciences, Graduate School of Biomedical Sciences, Nagasaki University, 1-7-1 Sakamoto, Nagasaki City 852-8501, Japan; bb55323810@ms.nagasaki-u.ac.jp (M.T.M.); uematsu@nagasaki-u.ac.jp (M.U.); yasserhelmy@nagasaki-u.ac.jp (Y.H.M.); maok@nagasaki-u.ac.jp (M.K.); tang-dy@nagasaki-u.ac.jp (D.T.); akio.oishi@nagasaki-u.ac.jp (A.O.); tkitaoka@nagasaki-u.ac.jp (T.K.); aohira@med.shimane-u.ac.jp (A.O.); 2 Department of Neurology and Strokology, Nagasaki University Hospital, 1-7-1 Sakamoto, Nagasaki City 852-8501, Japan; 3 Department of Neurology, Sasebo Chuo Hospital, 15 Yamatocho, Sasebo City 857-1195, Japan; shinkeinaika@gmail.com; 4 Department of Ophthalmology, Sasebo Chuo Hospital, 15 Yamatocho, Sasebo City 857-1195, Japan; 5 Department of Ophthalmology, School of Medicine, Shimane University, 89-1 Enya-cho, Izumo 693-8501, Japan

**Keywords:** corneal disorder, neuronal intranuclear inclusion disease, neurodegenerative diseases, neuro-ophthalmology, intranuclear inclusion bodies

## Abstract

*Background*: Neuronal intranuclear inclusion disease (NIID) is a progressive neurodegenerative disorder characterized by the formation of intranuclear inclusions in cells. Adult-type NIID usually develops in elderly patients with various clinical manifestations and is sometimes accompanied by ocular symptoms. A case of adult-onset NIID with early and unique manifestations, including a progressive corneal defect and retinal changes, which are concerning at a young age, is reported. *Case Presentation*: A 29-year-old woman with adult sporadic NIID presented to our department with a progressive corneal disorder. Her neurological symptoms started at the age of 22 years, and she was diagnosed with NIID by skin biopsy and genetic testing. Ocular examination revealed bilateral corneal superficial punctate keratitis, right corneal opacity, decreased vision, nocturnal lagophthalmos, and early retinal changes. Corneal nerve fiber atrophy was detected by in vivo confocal microscopy. With a Cochet–Bonnet aesthesiometer, the progression of NIID and decreased corneal sensation were confirmed. Findings consistent with neurotrophic keratitis and keratoconjunctivitis due to nocturnal lagophthalmos were both suggested as being complications of her underlying NIID. Treatment with punctal plugs, sodium hyaluronate eye drops, diquafosol sodium eye drops, systemic and local antivirals, and local steroid medications resulted in the gradual improvement in the irregularity and opacity of the epithelium. *Conclusions:* NIID may lead to neurotrophic keratopathy due to impairment of the corneal sensory nerves. Nocturnal lagophthalmos is a remarkable finding in a case of NIID. The findings in the present case highlight the complex and multifaceted nature of NIID, with neurological and ocular manifestations requiring a multidisciplinary approach to management.

## 1. Introduction

Neuronal intranuclear inclusion disease (NIID) is a rare neurodegenerative disorder characterized by the presence of intranuclear inclusions of unknown origin. Eosinophilic intranuclear inclusions are observed in the central and peripheral nervous systems, as well as in the visceral organs [1]. NIID is caused by the expansion of GGC repeats located in the 5′ UTR of the *NOTCH2NLC* (*N2C*) gene. It has been discovered that these repeats are present in a small upstream open reading frame (uORF) known as uN2C, which leads to their translation into a protein containing polyglycine, which is called uN2CpolyG [2]. NIID presents with various clinical forms and affects multiple body systems. The neurological manifestations include cognitive dysfunction, peripheral neuropathy, dyskinesia, paroxysmal symptoms, and autonomic dysfunction (Table 1) [1].

NIID is classified based on various factors such as family history, age of onset, clinical presentation, and the course of the disease. Depending on their family history, patients can be classified into sporadic and familial groups [1]. The disease can be categorized into infantile, juvenile, and adult forms based on age at the time of onset. The adult form mostly manifests after the age of 50 years. In East Asia, most of the cases of NIID are diagnosed as the adult form [3,4]. Based on the initial presentation and main symptomology, NIID is classified into four or five subgroups, such as dementia-dominant, movement-disorder-dominant, paroxysmal-symptom-dominant, muscle-weakness-dominant, and autonomic-dysfunction-dominant type [1,5]. Depending on the progression of the disease, NIID may be either episodically progressive or slowly progressive [4].

The diagnosis of NIID is confirmed by the presence of repeated GGC expansion in the 5′UTR of the *NOTCH2NLC* gene in skin biopsies [6]. These skin biopsies also show eosinophilic p62-positive intranuclear inclusions in the sweat gland cells and dermal adipocytes. Brain magnetic resonance imaging (MRI) is also used for diagnosis, particularly diffusion-weighted imaging (DWI). Curvilinear lesions in the corticomedullary junction on DWI are the best biomarkers for diagnosing NIID with high specificity (98.4%) and sensitivity (88.2%) [7].

NIID shows several ocular and neurological manifestations, which are summarized in (Table 1) [1,3,8,9].

A case of the adult form of NIID with the early onset of neurological and ocular manifestations is presented. The patient’s main symptom was recurrent corneal opacity with bilateral decreased corneal sensation. She also was found to have nocturnal lagophthalmos, which, to the best of our knowledge, has not been previously reported.

NIID cases were previously considered rare. However, more cases have been identified in clinical practice due to the availability of skin biopsy and gene analysis. The lack of diagnostic criteria and staging for this disease and its ophthalmological complications make it important to report any new symptoms or signs to effectively diagnose and manage such cases. Reporting the new and severe neurological and ocular manifestations in a young adult will contribute to the effective management of such cases, diagnosis, and future staging of the disease.

## 2. Case Presentation

A 29-year-old woman complained of bilateral blurred vision and foreign body sensation. She visited the ophthalmology clinic at Sasebo Chuo Hospital, where she was diagnosed with filamentous keratitis, and she was subsequently prescribed sodium hyaluronate and rebamipide eye drops. Two months later, she was referred to Nagasaki University Hospital due to the worsening of her corneal lesions without any pain.

Her history included a diagnosis of NIID. Her initial symptoms started at the age of 22 years with recurrent headaches and vomiting. Brain MRI showed hyperintensity on DWI and T2-weighted imaging in the corticomedullary border of both cerebral hemispheres (Supplementary Figure S1A,B), indicating progressive leukoencephalopathy. Genetic testing was negative for fragile X syndrome and the related genetic abnormalities, but it was positive for the GGC repeats on the *NOTCH2NLC* gene, which indicated NIID. A skin biopsy showed that there were many p62-positive intranuclear inclusions in the skin fibroblasts, sweat gland cells, and adipocytes (Supplementary Figure S1C–E). The definitive diagnosis was made based on the neurological symptoms, brain imaging, and genetic testing. She had no relevant family history and negative consanguinity.

On ocular examination, bilateral corneal epithelial and superficial stromal infiltrations, which were positive for fluorescein dye staining mainly in the palpebral fissure area and were accompanied by bulbar conjunctival injection, especially in the inferior part, were found. Her family reported that she kept both eyes open while sleeping, which suggested nocturnal lagophthalmos. The Schirmer test was performed without anesthesia, resulting in 20 mm in the right eye and 30 mm in the left eye. The corneal sensation test with a Cochet–Bonnet aesthesiometer was 35 mm (55 mg) for the right eye and 55 mm (24 mg) for the left eye (the average value for 30- to 40-year-old persons is ≤19 mg), which indicated bilateral decreased corneal sensation. No abnormal findings were apparent in the ocular media or the fundus, with normal intraocular pressure and BCVA. BCVA was 0.5 and 0.8 for the right and left eyes, respectively. A diagnosis of keratoconjunctivitis due to nocturnal lagophthalmos was made, and punctal plugs were inserted into the lower lacrimal puncta of both eyes. Ofloxacin eye ointment, 0.3%, administered once before sleep was also added. However, the patient was unable to attend her follow-up visits and stopped ocular treatment.

**Figure 1:**
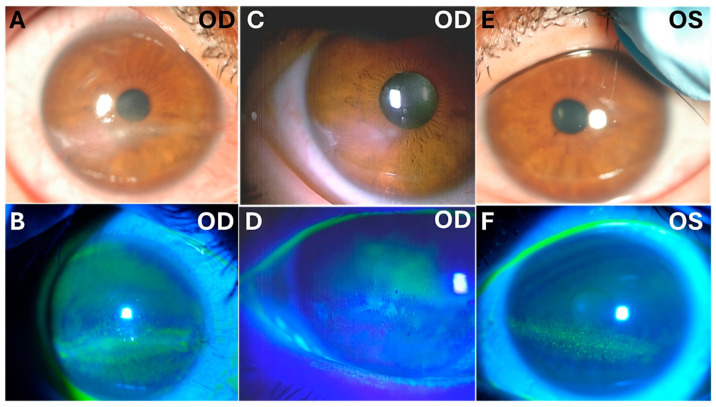
Slit-lamp and fluorescein dye examinations of the right and left corneas. (**A**) Slit-lamp microscopy shows right corneal opacity, infiltration, and pannus invasion; (**B**) fluorescein dye shows the right corneal opacity, which is more prominent in the palpebral fissure area; (**C**) slit-lamp microscopy of the right cornea after treatment shows decreased opacity and healing pannus; (**D**) right corneal fluorescein dye after treatment shows decreased corneal opacity; (**E**) slit-lamp microscopy shows that the left cornea is less affected than the right one; (**F**) fluorescein dye shows left corneal epithelial damage in the palpebral fissure area.

**Figure 2:**
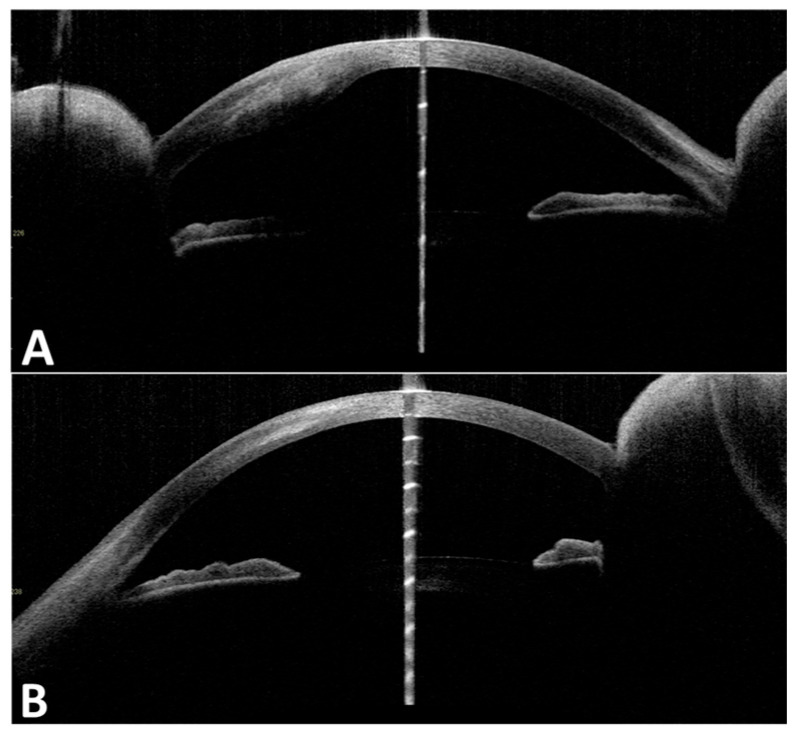
AS-OCT before and after treatment. (**A**) Right eye AS-OCT before treatment shows infiltration of the epithelium, subepithelium, and stroma, and corneal opacity (interstitial keratitis); (**B**) right eye AS-OCT after treatment shows decreased opacity.

**Figure 3:**
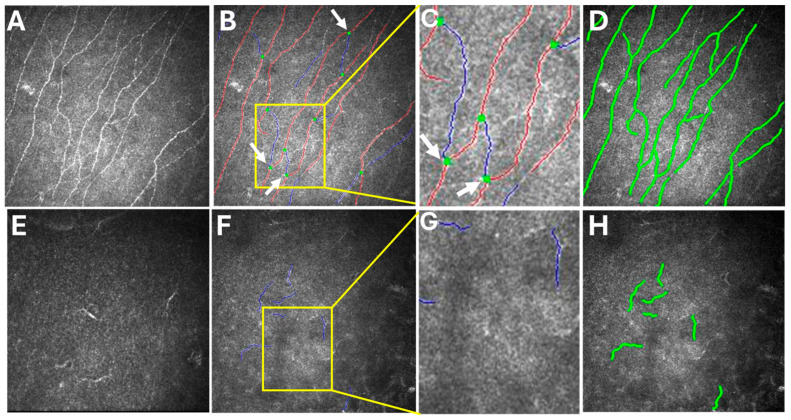
IVCM and its ACCMetrics interpretation comparing the present case with a normal person. (**A**) Normal person’s IVCM image; (**B**) illustrative representation of (**A**) image marked automatically with ACCMetrics (red: main corneal nerve fiber, blue: corneal nerve branch, green dot (white arrows): branch point; (**C**) a magnified inset of the image (**B**—yellow outlined box) showing the normal corneal innervation; (**D**) nerves detected automatically with ACCMetrics when analyzing image (**A**); (**E**) present case’s original IVCM image of the right eye; (**F**,**H**) illustrative representation of (**E**) image marked automatically with ACCMetrics, which shows a marked decrease in corneal innervation; (**G**) a magnified inset of the image (**F**—yellow outlined box) showing a lack of the corneal innervation compared to the normal person.

One year later, the patient returned with progressive blurred vision and decreased vision in her right eye. On examination, her intraocular pressure in both eyes was normal. Slit-lamp microscopy showed right corneal epithelial, subepithelial, and stromal infiltration with vascularization and ulceration (Figure 1). In contrast, her left eye showed superficial punctate keratitis with no opacities. The Cochet–Bonnet aesthesiometer showed no sensation at all in the right cornea and 25 mm (108 mg) in the left cornea, which indicated bilaterally reduced corneal sensation. Anterior segment optic coherence tomography (OCT) showed corneal epithelial and stromal opacity and thickening that indicated interstitial keratitis in the right eye (Figure 2). In vivo confocal microscopy (IVCM) showed a bilateral decrease in corneal innervation (Figure 3). IVCM images that showed the corneal sub-basal nerves were subsequently analyzed using ACCMetrics (version 3.0 for Windows, June 2015) under a license approved by Prof. Rayaz A. Malik (WCM-Q, Doha, Qatar), which is a fully automated image analysis software package (Figure 3) [3,10,11,12]. Corneal nerve fiber density (CNFD) was 0.0 fibers/mm^2^, corneal nerve branch density (CNBD) was 0.0 branches/mm^2^, corneal nerve fiber length (CNFL) was 5.15 mm/mm^2^, corneal nerve fiber area (CNFA) was 0.004 mm^2^/mm^2^, and corneal nerve fiber width (CNFW) was 0.025 mm/mm^2^ in the right eye.

With ACCMetrics analysis, the red line represents the main corneal nerve fiber, while the blue line represents a corneal nerve branch originating from the main corneal nerve fiber. Additionally, the green dot marks the point at which the corneal nerve branch originates from the main corneal nerve fiber. This green dot distinguishes branches from subbranches, which are small nerve fibers that arise from the branches rather than the main nerve fibers [3].

Fundus examination indicated that there was a bilateral mottled fundus appearance in the midperipheral retina that resembled the peau d’orange appearance in pseudoxanthoma elasticum and enlarged optic disc cupping (Supplementary Figure S2). Posterior segment OCT showed a bilateral mild disturbance of outer retinal integrity (Supplementary Figure S3). Electroretinography (ERG) showed subnormal rod and cone responses in both eyes (Supplementary Figure S4). The visual field measurements were not reliable due to the patient’s lack of concentration.

The right corneal opacity was scraped using a Golf Knife, and samples were submitted for culture and polymerase chain reaction (PCR) testing. The PCR results were negative for bacteria, viruses, and fungi. The results of culture testing showed that there was the growth of *Propionibacterium acnes*, possibly due to contamination. The patient was admitted to Nagasaki University Hospital and prescribed 0.3% gatifloxacin, 3% diquafosol sodium, 0.1% sodium hyaluronate, and 2% rebamipide eye drops TID for both eyes. Additional treatments due to the suspicion of herpetic keratitis included acyclovir eye ointment every 5 h, 0.1% fluorometholone eye drops QID for the right eye, and 500 mg oral valacyclovir hydrochloride for one week. Two weeks after admission, the patient was discharged from our department after partial improvement in the corneal opacity (Figure 2). At the time of discharge, th right BCVA was 0.7, and the left BCVA was 1.0.

The patient’s systemic comorbidities included epilepsy, cognitive impairment, ataxic-like speech, urinary and fecal incontinence, migraine, tension-type headache, peripheral neuropathy, hypertension, and bronchial asthma. She was using rizatriptan and valproate for migraine relief and prevention, respectively, as well as levetiracetam and divalproex sodium for epilepsy treatment. Nerve conduction studies (NCSs) showed decreased sensory conduction velocity (SCV) and nerve conduction velocity (NCV), which indicated polyneuropathy. Electroencephalography (EEG) showed no clear epileptic waves, but there were a significant number of slow waves throughout. Furthermore, the patient had a history of multiple admissions to the obstetrics and gynecology department due to severe hyperemesis gravidarum, threatened preterm labor, and abortion, which resulted in interruptions of the follow-up visits and ophthalmological treatments.

## 3. Discussion

This report presents the remarkable findings of a patient with NIID, adult form, and corneal opacity, bilateral nocturnal lagophthalmos, and early retinal changes. This patient presented with early-onset neurological and ocular manifestations compared with the manifestations normally found in the known previously reported adult-form NIID cases.

Most NIID cases with ocular symptoms have been shown to manifest after the age of 50 years, with the patients undergoing ophthalmological consultations at a later date [3,8,9,13]. However, the ocular symptoms in the present case started at the age of 28 years, with the subject immediately undergoing an ophthalmological evaluation. A review of the previous literature found only two cases of juvenile-onset NIID, with these patients being monozygotic twin sisters. In these previous cases, the symptoms started with dysarthria and nystagmus when the patients were 11 years old. The twins were only diagnosed after an autopsy was conducted in 1986, when they were 21 years old [13,14].

The bilateral recurrent corneal erosions in the present case were thought to be the result of neurotrophic keratitis and keratoconjunctivitis due to nocturnal lagophthalmos. The diagnosis of neurotrophic keratitis was confirmed by negative PCR and culture tests, with a decrease in corneal sensation confirmed by a Cochet–Bonnet aesthesiometer and IVCM findings. The neurotrophic keratitis was most likely due to the NIID. The NCS findings also supported the presence of polyneuropathy in NIID.

Nocturnal lagophthalmos was implicated in the keratoconjunctivitis in the present case. However, the cause of the nocturnal lagophthalmos could not be established despite being a remarkable manifestation.

The Cochet–Bonnet aesthesiometer showed deterioration in corneal sensation when comparing results from the first and second visits. This highlights the significance of the Cochet–Bonnet aesthesiometer in evaluating the progression of NIID and its corneal complications. If a Cochet–Bonnet aesthesiometer is not available, a cotton-tipped applicator could also be used, but the noncontact corneal aesthesiometer (NCCA) and corneal aesthesiometer Brill (CEB) are better alternatives.

The retinal findings included bilateral mottled fundus appearance in the mid-peripheral retina that resembled the peau d’orange appearance in pseudoxanthoma elasticum. The posterior segment OCT showed a mild disturbance of the outer retinal integrity, with the ERG showing decreased amplitude and subnormal rod and cone responses. These results suggest that these findings were indicative of the early retinal changes in a case of NIID, a finding that will be of help in the future staging of the disease. In addition, the present results also suggest that it is better to investigate the retina in NIID cases even without patient complaints.

However, there is no one theory that explains the ocular symptoms of NIID. Some researchers believe that the corneal symptoms are due to neurotrophic keratitis caused by autonomic nervous system dysfunction, affecting tear production and ocular homeostasis [9]. In our view, the corneal symptoms may be linked to the loss of sensory innervation in the cornea, leading to reduced corneal sensation and neurotrophic keratitis. Others believe that vision in NIID cases is affected by damage to the optic radiation, visual cortex, visual pathway, optic nerve, and retina, since intranuclear inclusions are found throughout the visual pathway [13].

Ocular symptoms can occur due to the direct involvement of inclusion bodies in the eye [13] or indirectly through the effects on the central and peripheral nervous systems [9,13].

The present case showed the early onset of the clinical symptoms that are associated with adult-form NIID. Previous studies reported that the mean (SD) age at the onset of adult NIID cases was 56.7 (10.3) years, with the most common initial symptom of NIID reported to be cognitive impairment [4,5,6,15]. However, the symptoms in the present case were sporadic, with an onset age of 22 years, and the initial symptoms were paroxysmal headache and vomiting.

In familial NIID cases, it has been reported that muscle weakness is seen most frequently, followed by sensory disturbance, miosis, bladder dysfunction, and dementia [15]. Despite being sporadic, the present patient showed similarities to familial cases, such as sensory disturbance, bladder dysfunction, and dementia. In the present case, there was obvious overlap between the known previous presentations of sporadic and familial adult NIID patients. The wide variety of clinical presentations of this disease makes it difficult to diagnose and classify.

The primary modality for the diagnosis of NIID in the present case was brain MRI, the findings of which were confirmed by the subsequent skin biopsy. Brain MRI findings are a useful screening tool in NIID. In cases with multiple or unexplained neurological symptoms, it is recommended that a detailed interpretation of the brain MRI be performed and that the possibility of NIID be considered.

NIID used to be difficult to diagnose, with definitive diagnoses only achieved after a skin biopsy and genetic testing. However, there has been a dramatic increase in the number of cases diagnosed with NIID when using skin biopsies and genetic testing [13,16].

It is our belief that the symptoms, examination, and investigation findings that were observed in the present case helped us to better understand the pathology and screening required for the early diagnosis of NIID cases. The pathology of keratitis in NIID cases may be related to a defect in corneal sensory innervation. This hypothesis is supported by the IVCM findings and the decreased corneal sensation that was confirmed by the Cochet–Bonnet aesthesiometer. We hope that these findings will be of help in the future staging of NIID, since we reported early changes in the retina, which are mild compared to those in NIID cases, and presented late with rod and cone dysfunction, retinal degeneration, and chorioretinal atrophy [8,13].

Given the presence of systemic comorbidities, it is crucial to consider the patient as having a multisystemic disease rather than being a case solely focused on a corneal disorder. It should be noted that during the patient’s stay in our department, she was found t unconscious once due to convulsions. These convulsions were treated, and she regained consciousness. This underscores the importance of comprehensive and vigilant management and cooperation with other departments.

## 4. Conclusions

NIID may present with ocular manifestations such as neurotrophic keratitis due to the loss of corneal sensation. Nocturnal lagophthalmos is a remarkable finding in a case of NIID. Retinal findings such as bilateral mottled fundus appearance in the mid-peripheral retina and disturbance of outer retinal integrity on posterior segment OCT, and ERG changes should be considered for the screening and staging of NIID cases. In addition, the Cochet–Bonnet aesthesiometer is a useful tool for testing corneal sensation in cases of NIID, and it can be used to monitor functional decline.

## Figures and Tables

**Table 1:** Neurological and ocular manifestations of NIID *.

Neurological NIID Manifestations	Ocular NIID Manifestations
Cognitive dysfunction	Oculogyric crisis
Movement disorders or dyskinesia	Reduced eye movements
Paroxysmal symptoms	Nystagmus
Peripheral neuropathy	Reduced best-corrected visual acuity (BCVA), especially in adult-onset NIID
Autonomic dysfunction	Rod and cone dysfunction
	Retinal degeneration
	Night blindness
	Miosis
	Ptosis
	Blepharospasm
	Neurotrophic keratitis

* NIID = neuronal intranuclear inclusion disease.

## Data Availability

The datasets generated and/or analyzed during the present study are available and will be published as Supplementary Information in Medicina. These include all raw data, processed data, and materials used in the research. The data will be accessible without restriction to ensure transparency and reproducibility of the results. Further inquiries can be directed to the corresponding author.

## Supplementary Materials

The following supporting information can be downloaded at: https://www.mdpi.com/article/10.3390/medicina60111730/s1, Figure S1: Brain MRI shows hyperintensities in the corticomedullary region, and the skin biopsy shows intranuclear inclusion bodies in the skin fibroblasts, sweat glands, and adipocytes. Figure S2: Fundus examination shows a bilateral mottled fundus appearance in the mid-peripheral retina resembling the peau d’orange appearance in pseudoxanthoma elasticum and bilateral optic disc cupping. Figure S3: Posterior segment optical coherence tomography (PS-OCT) shows a bilateral, mild disturbance of outer retinal integrity. Figure S4: Electroretinography (ERG) shows reduced amplitude with subnormal rod and cone responses in both eyes.

## Abbreviations

BCVA	Best-corrected visual acuity
DWI	Diffusion-weighted images
EEG	Electroencephalogram
ERG	Electroretinography
IVCM	In vivo confocal microscopy
MRI	Magnetic resonance imaging
NCS	Nerve conduction studies
NCV	Nerve conduction velocity
NIID	Neuronal intranuclear inclusion disease
OCT	Optical coherence tomography
PCR	Polymerase chain reaction
QID	Four times a day
SCV	Sensory conduction velocity
TID	Three times a day
