# Supplementary figures and images for: Neuronal Intranuclear Inclusion Disease with a Corneal Disorder: A Case Report
Source: Medicina (Kaunas). 2024 Oct 22;60(11):1730. doi: 10.3390/medicina60111730 (PMC11595896; doi:10.3390/medicina60111730)

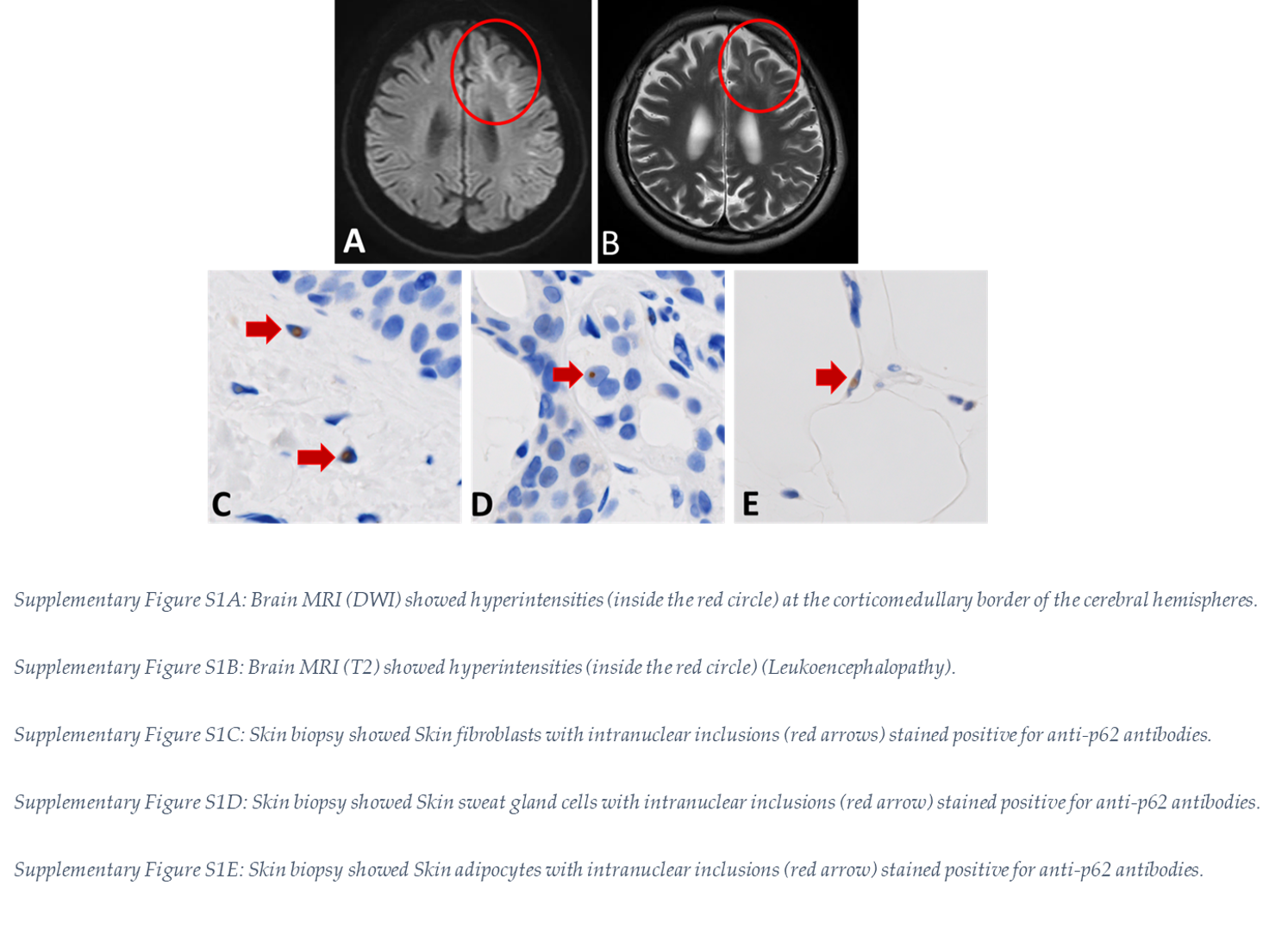

Supplement: Supplementary file 1 [file medicina-60-01730-s001.zip › Supplementary Figure S1.tif]

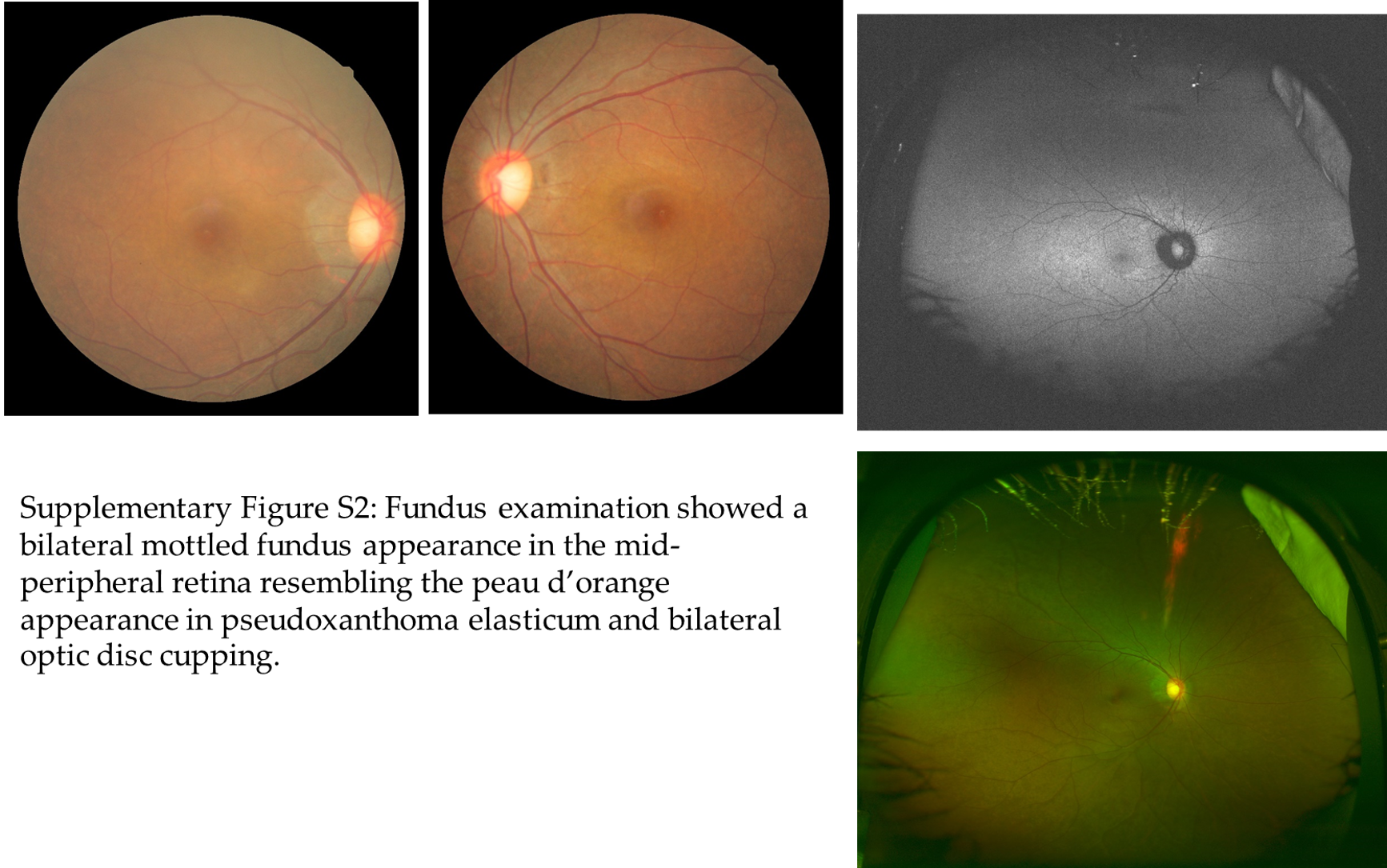

Supplement: Supplementary file 1 [file medicina-60-01730-s001.zip › Supplementary Figure S2.tif]

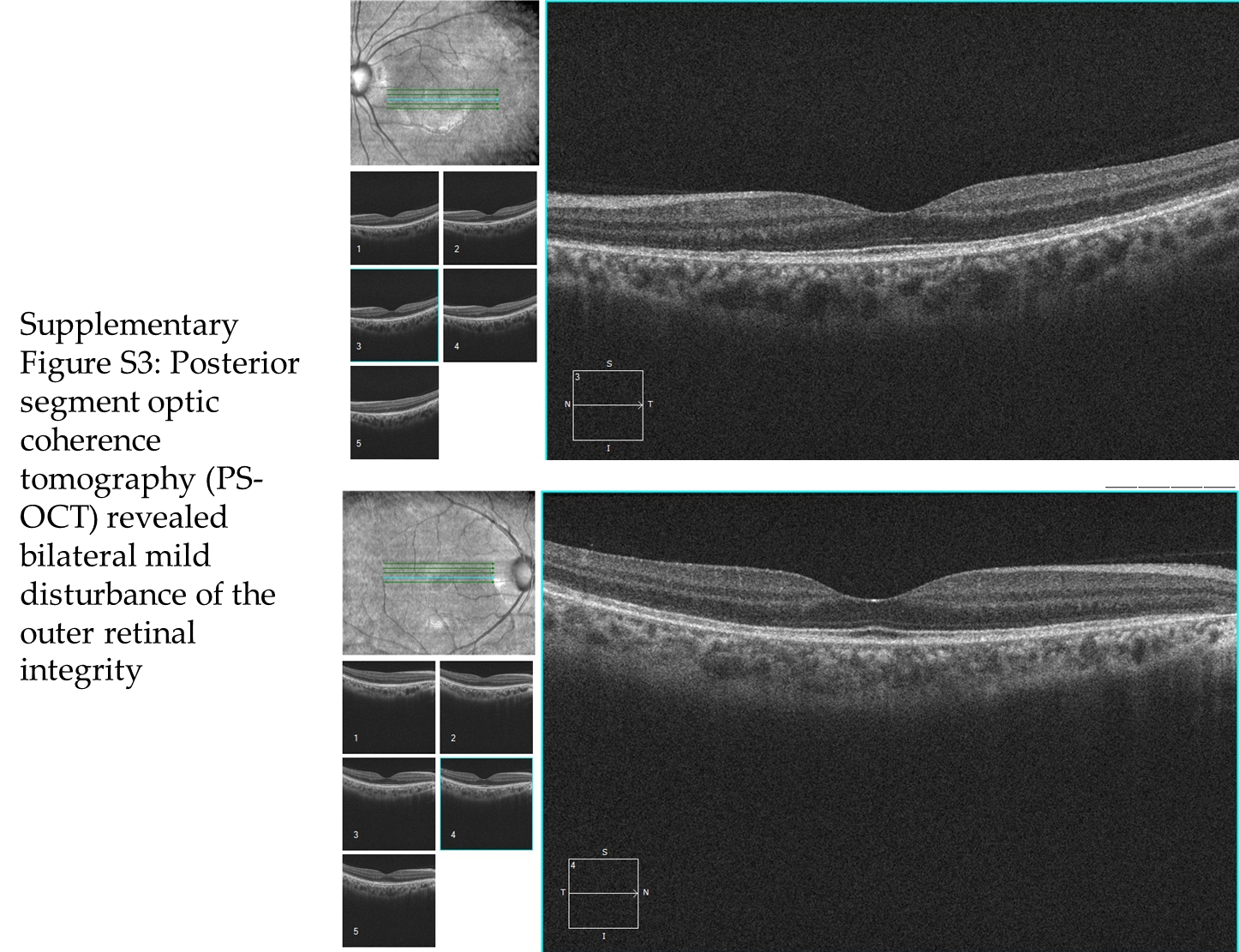

Supplement: Supplementary file 1 [file medicina-60-01730-s001.zip › Supplementary Figure S3.tif]

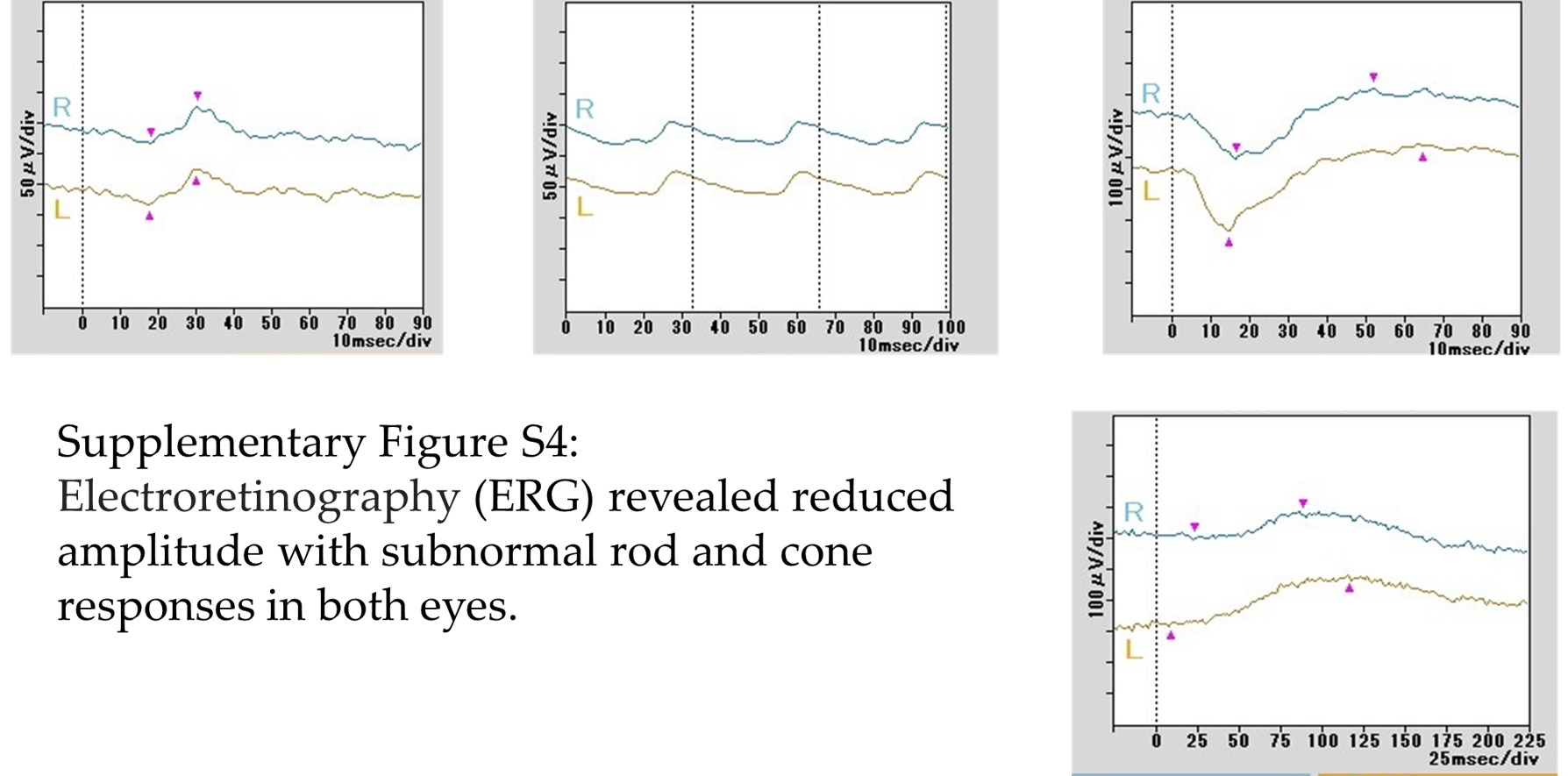

Supplement: Supplementary file 1 [file medicina-60-01730-s001.zip › Supplementary Figure S4.tif]
